# Multomics Analysis of the Characteristic Changes in Polyphenol Accumulation and Cell Wall Polysaccharide Remodelling During the Development of *Zingiber mioga* Roscoe Flower Buds

**DOI:** 10.3390/metabo16050316

**Published:** 2026-05-08

**Authors:** Chenglin Tang, Cheng Zhang, Xingyu Chen, Luolin Bao, Jiao Xie

**Affiliations:** 1 The key Laboratory of Environmental Pollution Monitoring and Disease Control, Ministry of Education, School of Public Health, Guizhou Medical University, Guiyang 561113, China; cl_tang520@163.com (C.T.); chengz76@gmc.edu.cn (C.Z.); cxy09102026@163.com (X.C.); 18385879968@163.com (L.B.); 2 Guizhou Crop Technology Extension Station, Agriculture and Rural Affairs Department of Guizhou Province, Guiyang 550001, China

**Keywords:** *Z. mioga*, polyphenolic compounds, cell wall polysaccharides, enzyme activities, gene expression, growth and ripening stage

## Abstract

**Background/Objectives**: At present, there are only a few studies on characteristic changes in polyphenols and cell wall polysaccharides and their correlations in *Z. mioga* flower buds during development. **Methods**: Polyphenols were analysed using ultra-performance liquid chromatography-tandem mass spectrometry (UPLC-MS/MS). Content and enzyme activities of cell wall components were examined using a microplate reader. Expression of genes related to these components was detected using de novo-Seq. **Results**: Most polyphenols accumulated significantly, with the highest levels being found in cyanidin-3-*O*-glucoside and epicatechin. PCA results show that changes in polyphenols were largely dependent on the germination and ripening stage, which might represent its specific period. Additionally, the increased flavonoid and anthocyanin fractions might be due to the up-regulated expression of *DFR*1/2, *ANS* and *BZl*. During development, PME, PG and *β*-galactosidase synergistically break down protopectin to soluble pectin; PME coordinates with cellulase in cellulose degradation, while xylanase dominates hemicellulose degradation. Lac collaborated with PME, PG and cellulase to regulate lignin synthesis. Key upregulated genes driving cell wall polysaccharide alterations include *PME*35, *PG* and *GAUT*7 for pectin metabolism, *CESA2/3* for cellulose synthesis, and *Lac*25, *POD*6/7/47/52 and *CCR*6 for lignin synthesis. Correlation analysis revealed that the synergistic effects of *p*-coumaric acid, chlorogenic acid, epicatechin, cyanidin-3-*O*-glucoside, peonidin-3-*O*-glucoside, protopectin, pectin, lignin and cellulose might be responsible for the sensory quality formation in *Z. mioga*. **Conclusions**: This study further investigates the binding mode of polyphenols and cell wall polysaccharides, providing a theoretical basis for understanding the development of sensory qualities in *Z. mioga* flower buds during growth and maturation.

## 1. Introduction

*Zingiber mioga* Roscoe (*Z. mioga*) is a perennial herb and vegetable of the genus Zingiber that is used as both medicine and food [1,2,3]. The flower buds of *Z. mioga*, which grow from underground rhizomes (with a shape similar to that of a pinecone), are the edible part of the plant and contain nutrients such as sugars, organic acids, amino acids and fatty acids, as well as bioactive ingredients such as polyphenolic compounds [3,4]. Notably, polyphenolic compounds with multiple phenolic hydroxyl groups are considered to be among the most abundant secondary metabolites synthesised in plant cells. Polyphenols such as anthocyanins (including mainly cyanidin-3-*O*-glucoside, petunidin-3-*O*-glucoside, pelargonidin-3-*O*-glucoside, peonidin-3-*O*-glucoside and delphinidin-3-*O*-glucoside), flavonoids (including mainly myricetin, rutin, isoquercitrin, epicatechin, epigallocatechin and dihydroquercetin) and phenolic acids (including mainly *p*-coumaric acid and chlorogenic acid) were the predominant polyphenols detected in *Z. mioga* flower buds [4]. However, most polyphenolic compounds found in vegetables are bitter and play important roles in flavour formation during growth and development [5,6]. Currently, *Z. mioga* is consumed in China mainly as bud shoots, which are cut into small pieces and eaten fresh in salads [3]. This suggests that polyphenolic compounds, which are closely linked to the formation of sensory quality, are particularly important for the flavour of *Z. mioga* flower buds during development.

The plant cell wall, as the outermost layer, consists of polysaccharides, phenolic compounds and structural proteins [7]. Cell wall polysaccharides and phenolics interact via multiple binding modes, including covalent and hydrogen bonds [8]. As a major class of plant-specific metabolites, phenolic acids play a critical role in cross-linking cellulose, hemicellulose and lignin within the plant cell wall network [9]. Accumulating evidence has demonstrated that fruit sensory properties, particularly masticatory texture, are predominantly determined by fruit texture characteristics, which rely heavily on the mechanical performance of parenchyma cell walls [10,11,12,13]. The texture (including firmness) is determined by the various components of the cell wall, such as pectin, cellulose, hemicellulose, and lignin, which form a cross-linked network structure through various chemical interactions (such as hydrogen bonding interactions, covalent bonds and hydrophobic forces), which has been widely documented in previous studies [14,15,16]. Additionally, the abundance and structural characteristics of texture-related cell wall components are dynamically modulated throughout plant growth and development [14,17,18]. Collectively, these findings indicate that cell wall polysaccharides were tightly associated with the formation of sensory quality and differentiation during plant development. For *Z. mioga* flower buds, development and maturation have been identified as the core factors governing their overall quality attributes [3,4,19]. Accordingly, dynamic alterations in cell wall polysaccharides are of great significance to the sensory flavour changes of *Z. mioga* flower buds during development.

To date, limited research has systematically explored polyphenolic compounds and cell wall polysaccharides, as well as their correlation, despite their crucial role in regulating the sensory flavour of *Z. mioga* flower buds during development. The main objective of this study is to provide a reference for further research on the influence of the developmental stage of *Z. mioga* flower buds on flavour quality by investigating the characteristic changes in the polyphenols and cell wall polysaccharides and their correlation during development. To understand the characteristic changes in polyphenols and cell wall polysaccharides and their correlation, the contents of polyphenol compounds were determined by ultra-performance liquid chromatography-tandem mass spectrometry (UPLC-MS/MS), the contents and enzyme activities of cell wall components were determined using a microplate reader, and the expression of genes related to these components was detected using de novo-Seq.

## 2. Materials and Methods

### 2.1. Plant Material

**Figure 1:**
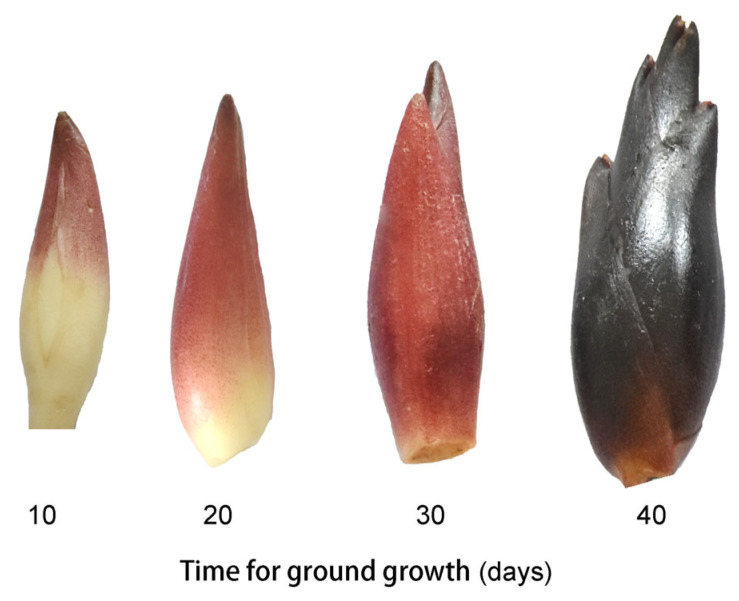
The flower bud progress diagram for *Z. mioga* during the development stage (GS1-GS4).

*Z. mioga* flower buds were obtained in 2020 from a vegetable garden in a mountainous area of Congjiang district (latitude 25° 50′ 33.8″ (N) and longitude 109° 7′ 18.3″ (E)), Guizhou, China. The buds that developed from underground rhizomes were collected at 10 d (the germination stage, GS1), 20 d (the budding stage, GS2), 30 d (the late developmental stage, GS3) and 40 d (the maturity stage, GS4) of growth (Figure 1). The samples of *Z. mioga* flower buds collected were identified by plant experts, and their appearance and morphology were consistent with those described by Deng et al. (2022) and Wei et al. (2023) [3,20]. Fifteen plants were selected as one replicate, and a total of forty-five *Z. mioga* flower buds at each developmental stage were collected, for a total of three replicates for each stage. The samples collected were uniform in terms of maturity (the maturity-related indicators, i.e., the soluble solid content, titratable acid content and solid-to-acid ratio, differed significantly between the stages, with the values shown in Figure S1 and assayed as described in Section 2.3), size, colour and appearance, and the samples were free from pests, diseases and mechanical damage. The collected samples were returned to the laboratory within two hours. They were then washed with tap water, air-dried at room temperature for 30 min and stored at −80 °C for two days prior to further analysis.

### 2.2. Chemicals and Reagents

Among the analytical reagents used in this study, carbazole and sodium hypochlorite were purchased from Shanghai Adamas Reagent Co., Ltd. (Shanghai, China); anhydrous ethanol and n-hexane were obtained from Tianjin Fuyu Fine Chemical Co., Ltd. (Tianjin, China); sodium acetate was purchased from Aladdin Biochemical Technology Co., Ltd. (Shanghai, China); acetyl bromide, bromothymol blue, *p*-nitrophenol, sodium acid phosphate, anhydrous sodium hydrogen phosphate, crosslinked polyvinylpyrrolidone, and ethylenediamine tetra-acetic acid were obtained from Maclin Biochemical Technology Co., Ltd. (Shanghai, China); sulfuric acid was purchased from Chongqing Chuandong Chemical Co., Ltd. (Chongqing, China); acetic acid was manufactured by Sichuan Xilong Science Co., Ltd. (Sichuan, China); nitric acid was obtained from Taicang Hu Test Reagent Co., Ltd. (Jiangsu, China); anthrone was obtained from Shanghai Yuanye Biotechnology Co., Ltd. (Shanghai, China); Dinitrosalicylic acid reagent and guaiacol were purchased from Soleibao Biotechnology Co., Ltd. (Shanghai and Beijing, China); polyvinylpyrrolidone, *p*-nitrophenyl-*β*-*D*-galactoside and *D*-(+) galacturonic acid were manufactured by Sigma-Aldrich and purchased from Shanghai Yuanye Bio-Technology Co., Ltd. (Shanghai, China); anhydrous sodium carbonate and sodium carboxymethylcellulose were obtained from Tianjin Yongda Chemical Reagent Co., Ltd. (Tianjin, China); and D-xylose was obtained from Dr. Ehrenstorfer GmbH (Augsburg, Germany). The authentic standard polyphenolic metabolites (≥95.0% pure) shown in Table S1 were obtained from Sigma-Aldrich (St. Louis, MO, USA). Merck (Darmstadt, Germany) provided HPLC-grade methanol and acetonitrile.

### 2.3. Determination of Soluble Solids and Titratable Acid

The soluble solid content was determined at room temperature using a PAL-1 digital refractometer (Atago, Tokyo, Japan). The titratable acid content was determined according to the method of Ao (2020) [21]. The solid-to-acid ratio was calculated as the ratio of soluble solids to titratable acid and is expressed as a percentage.

### 2.4. Extraction and Determination of Individual Polyphenols

Whole-cell extraction of polyphenolic compounds from the flower buds of *Z. mioga* during development was performed as previously described, with some modifications [22,23,24,25]. The preserved and lyophilised samples were ground at 30 Hz for 1.5 min. One hundred milligrams of powdered sample was extracted with 1.0 mL of methanol by vortexing for 1 min, sonication for 30 min and finally centrifugation at 11,190× *g* for 10 min at 4 °C. Prior to UPLC-MS/MS analysis, the supernatant was filtered through a 0.2 µm filter. The filtered extracts containing polyphenolic compounds were stored in injection vials until UPLC-MS/MS analysis.

The polyphenolic compounds were analysed using a UPLC-ESI-MS/MS system with an AB SCIEX Triple Quad 4500+ mass spectrometer equipped with an ESI turbo ion spray interface. The system was operated in positive and negative ion mode. The UPLC system was equipped with a Waters ACQUITY BEH C18 column (3.0 mm × 100 mm, 1.7 µm). Isocratic elution was carried out using a mobile phase comprising eluent A (pure water with 0.1% formic acid) and eluent B (methanol with 0.1% formic acid), with the ratio of 1:1. The injection volume and flow rate were 4 µL and 0.35 mL/min, respectively. The column temperature was maintained at 40 °C.

Linear ion trap (LIT) and triple quadrupole (QQQ) scans were performed on a QTRAP, QTRAP^®^ 6500+ UPLC-MS/MS system operated by Analyst 1.6.3 software (Sciex, Framingham, MA, USA), and the polyphenolic compounds were analysed. The ESI source operating parameters were as follows: ion source, ESI+; source temperature, 500 °C; ion spray (IS) voltage, 4500 V (positive mode) and −4500 V (negative mode); collision gas (CAD), 9; and curtain gas (CUR), 25 psi. Quantitative analysis of all the polyphenolic compounds was carried out using MultiQuant 3.0.3 software (Sciex, Framingham, MA, USA). QQQ scans were performed in multiple reaction monitoring (MRM) mode. The MRM transition parameter optimisations included DP and CE values for each compound obtained in positive or negative ion mode. Polyphenols were tentatively identified by comparing the spectral characteristics with those of authentic standards, as shown in Table S2, and quantified by the external standard method with reference to Kropek et al. (2023) and Liu et al. (2024) [24,26].

### 2.5. Measurement of Polysaccharide Components and Related Enzyme Activity in the Cell Wall

#### 2.5.1. Polysaccharide Components in the Cell Wall

The main cell wall components, including protopectin, soluble pectin, cellulose, hemicellulose and lignin, were analysed using a microplate reader (Thermo Fisher Scientific, Inc., Waltham, MA, USA). Protopectin and water-soluble pectin were extracted and quantified on the basis of methods published by Bu et al. (2013) and Cárdenas-Coronel et al. (2016) [27,28]. The pectin content was calculated using a standard curve (y = 0.0067x − 0.0008, R^2^ = 0.9994) generated with galacturonic acid and is expressed as mg·g^−1^ fresh weight (FW). Cellulose was extracted and its content was determined in accordance with the procedures described by Bu et al. (2013) [27]. Glucose levels were calculated on the basis of the absorbance of the supernatant at 620 nm and the standard curve of purified glucose (y = 0.006x − 0.0011, R^2^ = 0.9991) and then converted to the corresponding levels of cellulose using a coefficient of 0.9; the values are expressed as mg·g^−1^ FW. Hemicellulose was extracted and its content was measured according to the method reported by Tang (2015) [29]. Using *D*-xylose (y = 0.0153x + 0.0013, R^2^ = 0.9995) as the standard, the absorbance of the supernatant was measured at 660 nm, and the content of *D*-xylose in the sample was calculated and then multiplied by the coefficient 0.9 to determine the hemicellulose content (in mg·g^−1^ FW). The lignin content, expressed as ΔOD_280_ kg^−1^, was determined by measuring the absorbance at 280 nm as described by Deng et al. (2015) [20].

#### 2.5.2. Activities of Enzymes Associated with Polysaccharide Components

The activities of enzymes associated with pectin, including pectin methylesterase (PME) and polygalacturonase (PG), were analysed using a microplate reader (Thermo Fisher Scientific, Inc., Waltham, MA, USA). PME was extracted and its activity was determined as described by Ren et al. (2020) and is expressed as ΔOD_620_ min^−1^·g^−1^ protein [30]. PG activity was determined by measuring the absorbance at 540 nm and using a standard curve (y = 0.0158x + 0.0385, R^2^ = 0.9991) prepared with *D*-(+) galacturonic acid and is expressed as μg·g^−1^·min^−1^ protein according to the extraction and measurement methods of Zhang et al. (2010) and Zhao (2017) [31,32]. Cellulase was extracted and its activity was measured according to the method of Lieng-Hong et al. (1999) at 620 nm using a standard curve (y = 0.0181x + 0.316; R^2^ = 0.9992) prepared with glucose and is expressed as μg∙g^−1^∙min^−1^ protein [33]. The activity of *β*-galactosidase was determined at 400 nm using *p*-nitrophenyl-*β*-*D*-galactoside as a standard (y = 0.0039x + 0.032, R^2^ = 0.9997) and is expressed as μg∙g^−1^∙min^−1^ protein according to the extraction and measurement methods of Li (2016) [34]. The activity of xylanase was assayed at 540 nm using xylose as the standard (y = 0.0299x − 0.0228, R^2^ = 0.9997) and is expressed as μg∙g^−1^∙min^−1^ protein according to the extraction and measurement method of Tang (2015) [29]. The activity of peroxidase (POD) was calculated on the basis of the absorbance at 470 nm and is expressed as ∆OD_470_ g^−1^·min^−1^ protein according to the extraction and measurement methods of Ao (2020) [21]. The activity of laccase (Lac) was determined at 420 nm and is expressed as ∆OD_420_ mL^−1^∙min^−1^ protein according to the extraction and measurement methods of Schroyen et al. (2017) [35].

### 2.6. De Novo-Seq Analysis

Total RNA was extracted from *Z. mioga* flower buds grown in the ground for 10, 30 and 40 d using a TRIzol reagent (Invitrogen, Carlsbad, CA, USA), according to the manufacturer’s instructions. The quantity and purity of the total RNA were analysed with the 2100 Bioanalyzer and RNA 1000 Nano LabChip Kit (Agilent Technologies, Inc., Santa Clara, CA, USA), with the RNA integrity number (RIN) being >7.0, and three biological replicates being included for each sample group. Poly(A) RNA was purified from total RNA (5 μg) by two rounds of purification using poly-T-oligo magnetic beads to remove rRNA. After purification, divalent cations were applied at 95 °C for 3 min to fragment the mRNA into small pieces. The cleaved RNA fragments were then subjected to reverse transcription to produce cDNA. *E. coli* DNA polymerase I, RNase H and Dutp were subsequently used to generate U-tagged second-strand DNA from the cDNA. An A base was subsequently added to the blunt ends of each strand, followed by ligation to a modified Illumina multiplexed barcoding adapter containing custom unique molecular identifiers to minimise sequence bias and amplification noise, and size selection was performed with AMPureXP beads. The ligated products were amplified by PCR after enzymatic treatment of the U-labelled second-strand DNA with heat-labile UDG under the following conditions: initial denaturation at 95 °C for 3 min; 8 cycles of denaturation at 98 °C for 15 s, annealing at 60 °C for 15 s, and extension at 72 °C for 30 s; and a final extension at 72 °C for 5 min. The average insert size for the final cDNA library was 300 bp (±50 bp). Finally, paired-end sequencing was performed on an Illumina HiSeq 4000 at LC Sciences (LC Bio, China) according to the manufacturer’s recommended protocol. NCBI_NR (ftp://ftp.ncbi.nlm.nih.gov/blast/db/FASTA/nr.gz, accessed on 5 December 2024), SwissProt (http://www.ExPASy.ch/sprot, accessed on 5 December 2024), EggNOG (http://eggnog6.embl.de, accessed on 5 December 2024), and Pfam (http://pfam.xfam.org, accessed on 5 December 2024) databases were used for alignment with the sequence reads with the help of the TopHat package. Salmon was used to determine the expression levels of unigenes by calculating the transcripts per kilobase of exon model per million mapped reads (TPM) [36,37]. The differentially expressed genes (Table S3) were selected using the following thresholds: absolute value of the fold change ≥ 2 and *p* ≤ 0.05. For *Z*. *mioga* flower bud samples from three biological replicates, the ratios of TPM values at 40 d of growth in the ground to those at 10 d and 30 d and at 30 d of growth to those at 10 d were used as the fold change values.

### 2.7. Statistical Analysis

Cluster heatmaps were constructed using R language (R version 4.2.0 and ComplexHeatmap 2.12.0). A Venn diagram was constructed using R (version 3.5.1). The stacked bar chart was constructed with R language (R version 4.1.2). Principal component analysis (PCA) was performed using R software (R i386 ver. 3.3.3). The cell wall polysaccharide fraction and enzymatic activity data were analysed by analysis of variance (ANOVA) using the SPSS 22.0 software package (Duncan’s multiple comparison method), and significant differences among the GS1-GS4 flower buds were identified at the *p* < 0.05 level. Bar graphs were constructed using GraphPad Prism 7 (GraphPad Software Inc., CA, USA). R version 4.1.3 was used to construct the correlation network heatmap. A combined heatmap was generated using MetWare Cloud, a free online data analysis platform (https://cloud.metware.cn, accessed on 6 January 2025).

## 3. Results and Discussion

### 3.1. Changes in Polyphenolic Compound Content and Related Gene Expression in Z. mioga During Growth and Ripening

#### 3.1.1. Changes in the Polyphenolic Compound Contents

**Figure 2:**
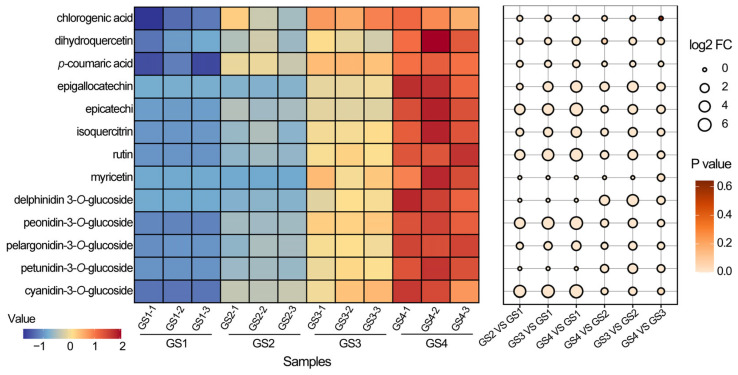
Flower bud progress diagram for *Z. mioga* during the development stage (GS1-GS4). Combinatorial heat map of changes in polyphenol compounds in *Z. mioga* flower buds during the development stage. Polyphenol compounds that met the criteria of *p* ≤ 0.05 were significantly different. GS1-1/1-2/1-3, the number of repeats at GS1 stage (10 days of growth or germinability); GS3-1/3-2/3-3, the number of repeats at GS3 stage (30 days growing or late developing); GS4-1/4-2/4-3, the number of repeats at GS4 stage (40 days of growth or ripeness).

**Figure 3:**
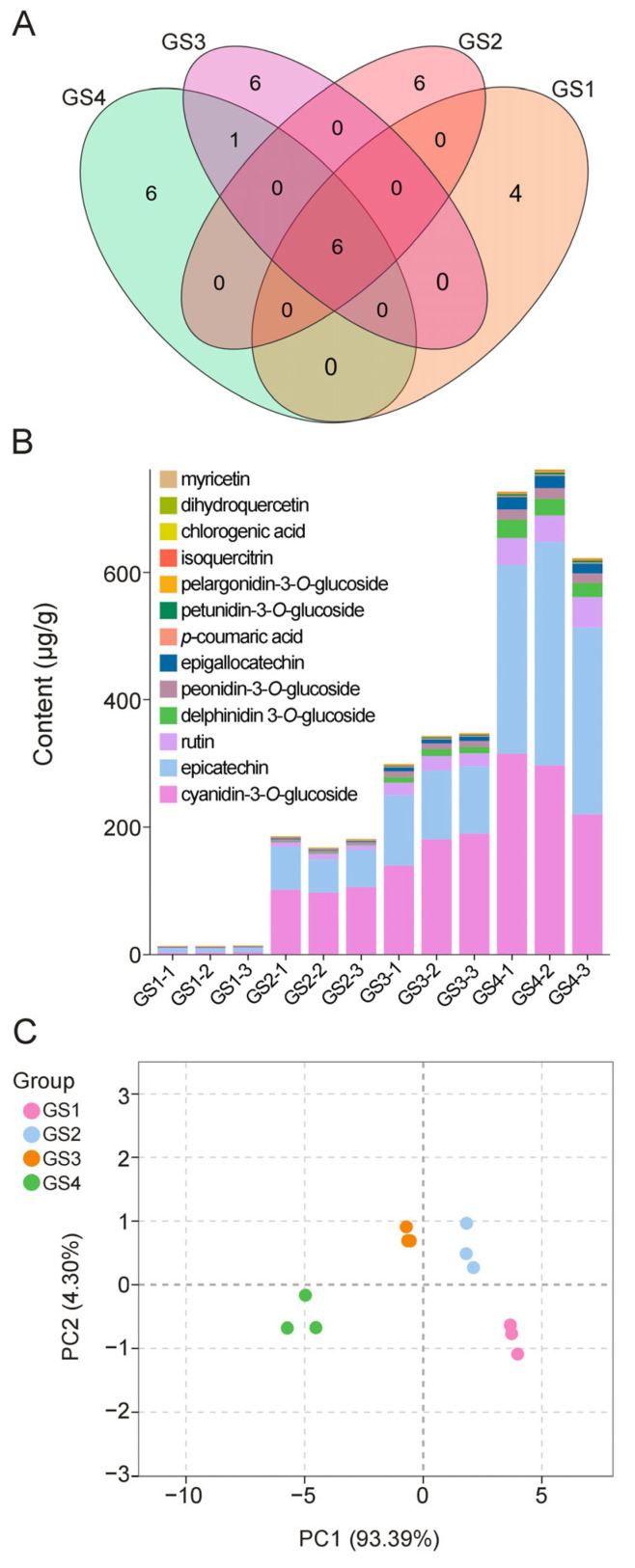
Multivariate statistical analysis of polyphenolic compounds in *Z. mioga* flower buds during the development stage. (**A**) Venn diagram; (**B**) stacked diagram for metabolite content; (**C**) principal component analysis (PCA). GS1-1/1-2/1-3, the number of repeats at GS1 stage (10 days of growth or germinability); GS2-1/2-2/2-3, the number of repeats at GS2 stage (20 days growing or budding); GS3-1/3-2/3-3, the number of repeats at GS3 stage (30 days growing or late developing); GS4-1/4-2/4-3, the number of repeats at GS4 stage (40 days of growth or ripeness).

According to the growth and development cycle of *Z. mioga* flower buds, four developmental stages (GS1–GS4) were examined. This study tentatively identified thirteen polyphenolic compounds using UPLC-MS/MS, including five anthocyanins (e.g., cyanidin-3-*O*-glucoside, petunidin-3-*O*-glucoside, pelargonidin-3-*O*-glucoside, peonidin-3-*O*-glucoside and delphinidin-3-*O*-glucoside) and eight flavonoids (e.g., epicatechin, rutin, epigallocatechin, isoquercitrin, chlorogenic acid, *p*-coumaric acid, dihydroquercetin and myricetin). Among these compounds, the presence of epicatechin and isoquercitrin was consistent with the reports by Liu et al. (2023) [4]. In particular, delphinidin 3-*O*-glucoside and myricetin were not found in the *Z. mioga* flower buds at the GS1 stage, or in myricetin at the GS2 stage (Figure 2 and Figure 3A). As shown in Figure 2 and Figure 3B and Table S1, the thirteen polyphenolic compounds tentatively identified in *Z. mioga* flower buds showed similar patterns during growth and ripening, gradually increasing over time until they reached their highest levels at the GS4 stage and their lowest levels at the GS1 stage. These results are consistent with those of Liu et al. (2023), whose study showed d that *Z. mioga* flower buds continuously accumulated anthocyanins and flavonoids during their growth, reaching maximum concentrations at maturity [4]. Significant differences in the levels of the thirteen polyphenolic compounds identified in *Z. mioga* flower buds were observed across the four developmental stages. However, except for chlorogenic acid, there was no significant difference in concentration between the GS4 and the GS3 stage. Among these, the highest levels throughout the entire developmental stage were recorded by cyanidin-3-*O*-glucoside (277.52 μg/g) and epicatechin (313.39 μg/g).

As demonstrated in Figure 3C, the findings of principal component analysis (PCA) revealed that the samples from the four stages grouped into four separate quadrants, indicating that flower buds at varying stages of development could be efficiently separated using the initial two principal components (PCs), with each growth stage exhibiting tight clustering. These results suggested that there were significant differences in polyphenol metabolite profiles across the whole stage and these changes largely depend on the growth and maturation stage. Overall, 97.69% of the total variation was explained by these two principal components (PC1 = 93.39% and PC2 = 4.30%). PC1 accounted for most of the variation and the data exhibited a single dominant dimension. This phenomenon, whereby a single principal component dominated while the explanatory power of subsequent components declined rapidly, was likely due to the strong autocorrelation generally existing among metabolites. In the PCA score plot, the first principal component ranged from the GS4 to the GS1 stage and was ordered from negative to positive on the X-axis. This plot shows that polyphenol levels varied throughout the growth period, consistent with the finding that polyphenolic compounds accumulated during development and maturation (Figure 2). The second principal component was plotted along the Y-axis. The GS2 and GS3 stages were in the positive quadrant, while the GS1 and GS4 stages were in the negative quadrant and in different regions. These results suggest that germination and maturity might be distinct periods of change in the polyphenolic compounds of *Z. mioga* flower buds. A study by Lee et al. (2016) indicates that the aqueous extract of *Z. mioga* exhibits antidiabetic properties owing to its high polyphenol content [1]. However, this study did not provide data on the dynamic changes in polyphenol levels. The main significance of our study is that it is the first to show stage-specific accumulation patterns of polyphenols in *Z. mioga* flower buds.

#### 3.1.2. Expression Levels of Genes Related to Anthocyanin Biosynthesis

**Figure 4:**
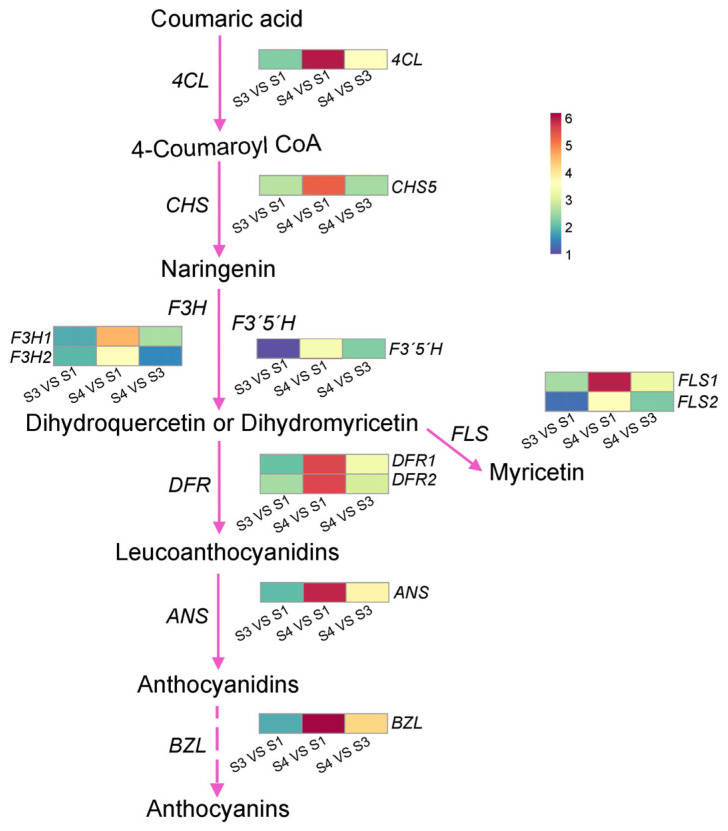
Schematic view of differentially expressed genes involved in anthocyanin synthesis pathway for *Z. mioga* flower buds growing at 0 d, 30 d and 40 d. Growth stage at 30 d VS at 0 d, S3 VS S1; growth stage at 40 d VS at 0 d, S4 VS S1; growth stage at 40 d VS at 30 d, S4 VS S3. The enzyme-encoding genes are abbreviated as follows: *4CL*, 4-coumarate-CoA ligase-like; *CHS*5, chalcone synthase 5; *F3H*1/2, mutant protein of flavanone-3-hydroxylase 1/2; *F3′H*, flavonoid 3′-monooxygenase; *F3′5′H*, flavonoid 3′,5′-hydroxylase 2-like; *FLS*1/2, flavonol synthase 1/2; *DFR*1/2, dihydroflavonol 4-reductase 1/2; *ANS*, anthocyanidin synthase; *BZl*, anthocyanidin 3-*O*-glucosyltransferase. These differentially expressed genes were selected by *p* value < 0.05 and ∣fold change∣ ≥ 2, and Tables S1–S3 show the detailed information.

Given the distinct colour variation observed in *Z. mioga* flower buds (Figure 1 and Figure 4 and S2), de novo-Seq analysis was used to screen the genes involved in anthocyanin metabolism. Two-by-two comparisons (GS4 vs GS1, GS4 vs GS3 and GS3 vs GS1) were conducted to identify the differential expression of genes, with thresholds of ∣log2-fold change∣ ≥ 1.00 and *p* < 0.05. The synthesis of anthocyanins primarily involves flavonoid biosynthesis and anthocyanin synthesis, with 250 and 96 genes identified in these processes, respectively (Figure S2). When GS1, GS2 and GS3 were compared, eight and three genes involved in flavonoid and anthocyanin synthesis were found to be expressed differently.

The initial reaction in the phenylpropanoid pathway involves anthocyanin biosynthesis, in which the 4-coumarate-CoA ligase-like (*4CL*) gene encoding coumarin-CoA is the primary gene responsible for catalysis [38]. As shown in Figure 4, the *4CL* expression increased throughout development, as seen in comparison of GS4 vs GS1, GS4 vs GS3 and GS3 vs GS1. During development, the expression levels of several flavonoid biosynthetic genes increased, including chalcone synthase 5 (*CHS* 5, for naringenin synthesis), as well as the flavanone-3-hydroxylase 1/2 (*F3H* 1/2), flavonoid 3′-monooxygenase (*F3′H*) and flavonoid 3′,5′-hydroxylase 2-like (*F3′5′H*) genes for dihydroflavonol synthesis. Among these genes, the upregulation of *F3H* 1/2 is consistent with the increase in dihydroquercetin content during development (Figure 2 and Table S1). In addition, the expression of flavonol synthase 1/2 (*FLS 1/2*), which catalyses the conversion of dihydroflavonol to flavonol in myricetin synthesis, increased during development. This finding aligns with the rise in myricetin content observed in developing *Z. mioga* flower buds (Figure 2 and Table S1). Furthermore, the expression levels of two key anthocyanin biosynthesis genes, dihydroflavonol 4-reductase 1/2 (*DFR*1/2) and anthocyanidin synthase (*ANS*), were also significantly upregulated during development. The gene responsible for converting anthocyanidin to anthocyanin in *Z. mioga* flower buds was mainly anthocyanidin 3-*O*-glucosyltransferase (*BZl*). This increased in expression as buds developed, coinciding with higher levels of specific anthocyanins such as cyanidin-3-*O*-glucoside, petunidin-3-*O*-glucoside, pelargonidin-3-*O*-glucoside, peonidin-3-*O*-glucoside and delphinidin-3-*O*-glucoside (Figure 2 and Table S1). In summary, developmental increases in flavonoid and anthocyanins resulted mainly from the higher expression of *DFR*1/2, *ANS* and *BZl*, confirming a gene-mediated, stage-specific accumulation pattern [4].

### 3.2. Changes in Polysaccharide Components and Related Enzyme Activity in the Cell Wall

#### 3.2.1. Changes in the Pectin Level and Associated Enzyme Activities

**Figure 5:**
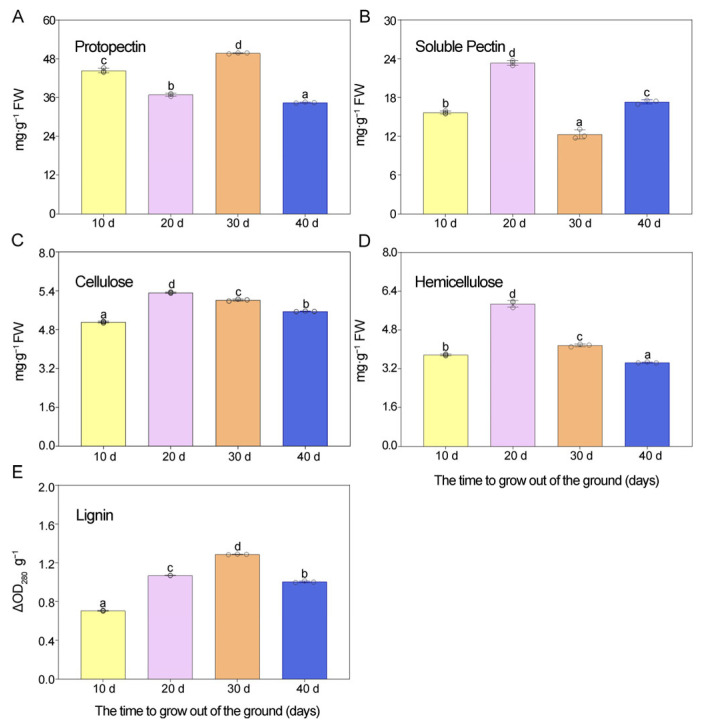
Changes in compositions of cell wall polysaccharides in flower buds of *Z. mioga* during development. Pectin, soluble pectin, cellulose, hemicellulose and lignin content levels are indicated by (**A**), (**B**), (**C**), (**D**) and (**E**) respectively. In each column, the mean value of the three replicates is shown, and the bars indicate standard errors. Using Duncan multiple comparisons, different lowercase letters in the same illustration indicate significant differences (*p* < 0.05) for different growth stages of *Z. mioga*.

**Figure 6:**
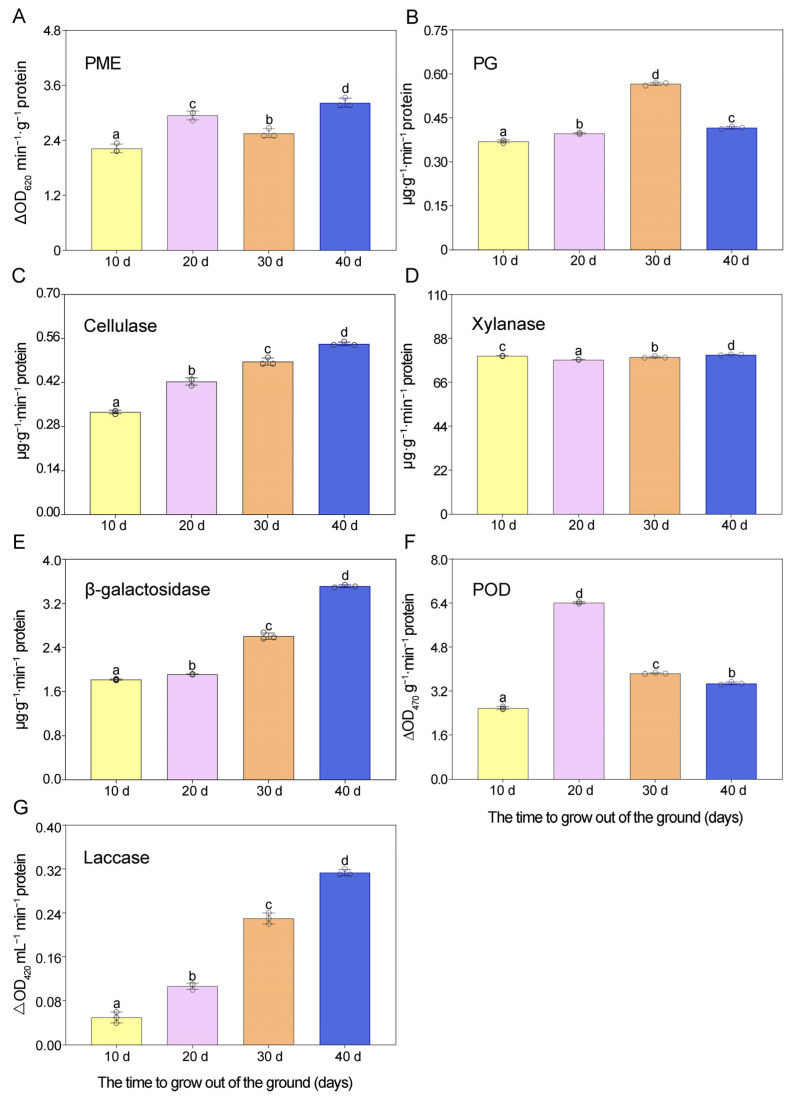
Changes in enzyme activities associated with cell wall polysaccharides in flower buds of *Z. mioga* during development. The activities of PME, PG, cellulase, xylanase, *β*-galactosidase, POD and laccase are marked with (**A**), (**B**), (**C**), (**D**), (**E**), (**F**) and (**G**) respectively. PME, pectin methylesterase; PG, pectin methylesterase; POD, peroxidase. In each column, the mean value of the three replicates is shown, and the bars indicate standard errors. Using Duncan multiple comparisons, different lowercase letters in the same illustration indicate significant differences (*p* < 0.05) for different growth stages of *Z. mioga*.

As shown in Figure 5A, there was a significant decrease in protopectin content, followed by an increase and then a final decrease. The highest level was detected at the GS3 stage (49.72 mg·g^−1^) and the lowest at the GS4 stage (34.45 mg·g^−1^). As shown in Figure 5B, the content of water-soluble pectin exhibited a significant increase, followed by a decrease, and then a final increase. The highest content was observed at the GS2 stage (23.35 mg·g^−1^) and the lowest at the GS3 stage (12.31 mg·g^−1^). Studies have shown that water-soluble pectin is converted from protopectin during the ripening process [39]. Therefore, these dynamic changes in the water-soluble pectin content of *Z. mioga* flower buds are consistent with the changes in protopectin content. The two main enzymes involved in pectin degradation are PME and PG (Figure 6A,B). PME catalyses the de-esterification of pectin during ripening, increasing the water solubility of pectin and thus creating conditions suitable for the PG catalysis [40]. PG catalyses the depolymerisation and solubilisation of pectin and plays important roles in its degradation and conversion [15]. During development and ripening, the activity of the PME in the *Z. mioga* flower buds first increased significantly, then decreased, and then increased again. The greatest PME activity was found at the GS4 stage (3.22 ∆OD_620_ min^−1^·g^−1^ protein), while the lowest activity was the GS1 stage (2.22 ∆OD_620_ min^−1^·g^−1^ protein). In addition, an evident trend of increased followed by decreased activity was detected for PG, with the highest activity occurring at the GS3 stage (0.68 µg·g^−1^·min^−1^ protein) and the lowest activity occurring at the GS1 stage (0.44 µg·g^−1^·min^−1^ protein). These results indicate that the combined effects of PG and PME resulted in the conversion of pectin in *Z. mioga* flower buds into soluble pectin.

#### 3.2.2. Changes in the Levels of Cellulose and Hemicellulose and the Associated Enzyme Activities

Cellulose plays a key role in maintaining the firmness of fruit and vegetables by supporting the cell wall skeleton. In other words, cellulose degradation leads to thinning of the cell wall and a decrease in its hardness [41,42,43]. As shown in Figure 5C, cellulose content initially increased significantly during development, before decreasing. The highest cellulose content was observed at the GS2 stage (5.55 mg·g^−1^), and the lowest at the GS1 stage (5.11 mg·g^−1^). The key enzyme involved in cellulose degradation is cellulase, which catalyses the continuous hydrolysis of amorphous cellulose into cellulosic oligosaccharides [29]. In this study, cellulase activity significantly increased during development (Figure 6C), with the highest activity (0.54 µg·g^−1^·min^−1^) observed at the GS4 stage. These results reveal a gradual decrease in the cellulose content from the GS2 to the GS4 stage in *Z. mioga* flower buds, coinciding with increased cellulase activity.

Hemicellulose and cellulose coexist in plant cell walls [29]. Hemicellulose is a major heteropolysaccharide made mainly of xylan and galactose [39]. The hemicellulose content initially rose and then fell during development (Figure 5D), peaking at the GS2 stage (5.88 mg·g^−1^). This pattern mirrors changes seen in thin-fleshed fruit like *Momordica charantia* [44]. In addition, as shown in Figure 6D,E, xylanase and *β*-galactosidase activities both increased later in development (Peaking at the GS4 stage, 79.84 and 3.51 µg·g^−1^ protein, respectively). These enzymes break down cell wall components [44], suggesting that their rising activity caused the observed decline in hemicellulose levels in *Z. mioga* flower buds. Other factors, such as the presence of inhibitors that interfere with hemicellulose biosynthesis, might also contribute.

#### 3.2.3. Changes in the Lignin Content and Associated Enzyme Activities

Lignin binds with carbohydrates like cellulose, hemicellulose and pectin to form a rigid network that strengthens plant cell walls [45]. In the *Z. mioga* flower, lignin content peaked at the GS3 stage (1.29 ∆OD_280_ g^−1^) and was lowest at the GS1 stage (0.71 ∆OD_280_ g^−1^), as shown in Figure 5E. Research has shown that high lignin levels increase hardness and reduce texture quality [46]. Therefore, the decrease in lignin content at the GS4 stage improved the quality of *Z. mioga* flower buds. The primary enzymes involved in lignin synthesis are POD and Lac. As shown in Figure 6F,G, POD activity rose sharply until the GS2 stage (6.41 ∆OD_470_ g^−1^·min^−1^ protein) and then declined, while Lac activity increased steadily throughout development, peaking at the GS4 stage (0.31 ∆OD_420_ mL^−1^·min^−1^ protein). These results suggest that the initial rise in lignin (from GS1 to GS2) was mainly driven by increased POD and Lac activity. Additionally, the further increase in lignin content at the GS3 stage was likely due to rising Lac activity. However, the drop in lignin content at the GS4 stage occurred despite high Lac activity, coinciding instead with falling POD activity. This suggests that while both enzymes contributed to lignin production, their POD activity patterns did not perfectly match lignin levels, possibly because they also served other functions like oxidative stress and pathogen defence [46,47].

#### 3.2.4. Correlation Analysis of Polysaccharide Components and Enzyme Activities in the Cell Wall

**Figure 7:**
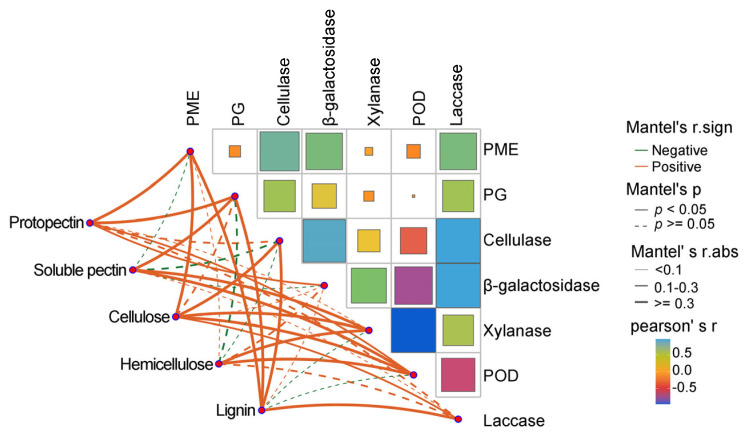
Correlation network heat map analysis between cell wall polysaccharide fractions and their enzymatic activities. Mantel’s r. sign, positive or negative correlation on the basis of the Pearson correlation coefficient; Mantel’s *p*, the *p*-value threshold in the Mantel test; Mantel’s r.abs, the absolute value threshold of correlation between two distance matrices in Mantel’s test; Pearson’s r, Pearson correlation coefficient. Pectin methylesterase, PME; polygalacturonase, PG; Peroxidase, POD.

Correlation analysis using the Mantel test and Pearson correlation coefficient revealed relationships between the polysaccharide component and associated enzyme activities during *Z. mioga* growth. As shown in Figure 7, the Mantel test was used to assess the relationships between cell wall polysaccharide components and the corresponding metabolic or synthetic enzymes. In this study, the negative correlations of the five polysaccharide components and the seven enzymes were not significantly different (*p* ≥ 0.05). Significant differences and strong positive correlations were observed between the protopectin content and the activities of PME, PG, *β*-galactosidase and POD; the soluble pectin content and the activities of PG, xylanase and POD; the cellulose content and the activities of PME, cellulase, xylanase, POD and Lac; the hemicellulose content and the activities of xylanase and POD; and the lignin content and the activities of PME, PG, cellulase and Lac. Notably, *β*-galactosidase activity correlated exclusively with protopectin content, consistent with its role in degrading pectin and hemicellulose [44], suggesting it is a key driver of pectin degradation.

Pearson’s correlation coefficient was used to analyse the correlations between the activities of the seven enzymes. As shown in Figure 7, the magnitude of the correlation coefficient is indicated by the size of the coloured box, where the colour indicates a positive or negative correlation. The *p* values obtained from the Pearson correlation analysis are given in Table S1. As shown in Figure 7 and Table S2, strong positive correlations were observed among the following: cellulase and Lac, *β*-galactosidase, PME and PG activities; Lac and *β*-galactosidase, PME and PG activities; and *β*-galactosidase and PME and PG activities. However, a strong and significant negative correlation was observed between POD activity and xylanase activity. Strong correlations were also detected between *β*-galactosidase activity and PME and PG activity, as well as between *β*-galactosidase activity and protopectin content. In summary, regarding pectin degradation, our results suggest that *β*-galactosidase appeared to participate in this process by interacting with PME and PG, which helps to maintain the quality of *Z. mioga* flower buds at the GS4 stage. For cellulose degradation, cellulase was the primary enzyme for cellulose breakdown, likely to work synergistically with PME. According to the hemicellulose degradation, xylanase was directly involved in hemicellulose breakdown. POD seemed to antagonise (negatively regulate) xylanase activity. Regarding to lignin synthesis, Lac was involved in lignin synthesis, likely cooperating with PME, PG, and cellulase. In summary, six enzymes (PME, PG, cellulase, xylanase, *β*-galactosidase and Lac) were closely linked to the degradation of cell wall polysaccharides. Consistent with prior studies, this research highlights that POD modulated hemicellulose degradation by antagonising xylanase, [4,39,44], reflecting dynamic changes during *Z. mioga* flower bud development.

#### 3.2.5. Expression of Genes Involved in Pectin, Lignin and Cellulose Metabolism

Differentially expressed genes related to cell wall polysaccharide metabolism were screened via pairwise comparisons (∣log2-fold change∣ ≥ 1.00 and *p* < 0.05) across three developmental stages (GS1, GS3 and GS4 stage) of *Z. mioga* flower buds (GS4 vs GS1, GS4 vs GS3 and GS3 vs GS1). In total, 179, 204 and 125 genes were annotated to pectin, cellulose and lignin metabolism, respectively, (Figure S3). In the GS4 vs GS1 comparison, five, five and six of the differentially expressed genes were linked to the three pathways separately: five, five and six were upregulated, while one, zero and two were downregulated. Notably, there was no difference in the expression of peroxidase 5 (*POD5*) or laccase 24 (*Lac*24) between the GS4 and GS3 samples. Similarly, peroxidase 15 (*POD5*), galacturonosyltransferase 7 (*GAUT7*), cellulose synthase 2/3 (*CESA*2/3), cinnamoyl-CoA reductase-like 6 (*CCR6*), pectin methylesterase 35 (*PME35*) and PG showed stable expression between the GS3 and GS1 stage.

**Figure 8:**
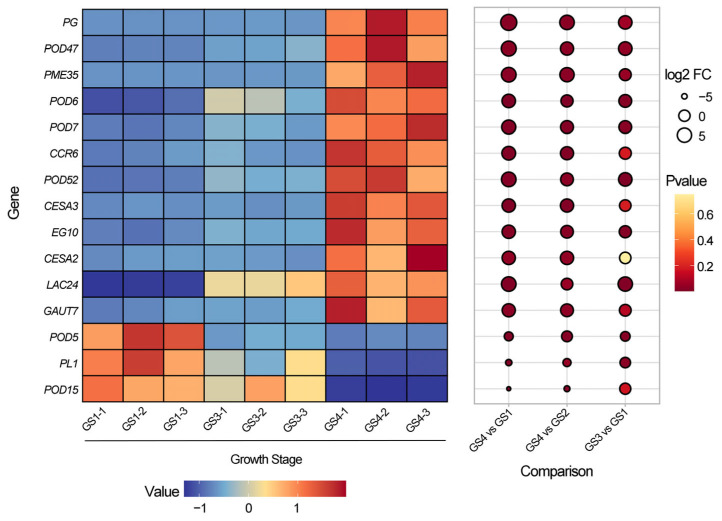
Combined heat map of the gene expression patterns involved in the metabolism of pectin, lignin, hemicellulose and cellulose in the flower buds of *Z. mioga* during development. Genes that met the criteria of ∣log 2-fold change∣ ≥ 1.00 and *p* ≤ 0.05 were differentially expressed. GS1-1/1-2/1-3, the number of repeats at GS1 stage (10 days of growth or germinability); GS4-1/4-2/4-3, the number of repeats at GS4 stage (40 days of growth or ripeness). Pectin methylesterase 35 (*PEM*35), the gene encoding pectin methylesterase; polygalacturonase (*PG*), the gene encoding polygalacturonase; pectate lyase 1 (*PL*1), the genes encoding pectate lyase; galacturonosyltransferase 7 (*GAUT7*), the genes involved in pectin biosynthesis; cellulose synthase 2/3 (*CESA*2/3) and endoglucanase 10 (*EN*10), the genes encoding enzymes for cellulose degradation; endoglucanase and cinnamoyl-CoA reductase-like 6 (*CCR*6), the genes encoding enzymes for lignin synthesis; peroxidase 5/6/7/15/47/52 (*POD*5/6/7/15/47/52) and laccase 24 (*Lac*24), the genes encoding enzymes for lignin degradation.

As shown in Figure 8, four pectin metabolism-related differentially expressed genes were detected in *Z. mioga* flower buds, including degradation genes [*PG*, *PME35* and pectate lyase 1 (*PL1*)] and the synthesis gene *GAUT7*. During development, *PG*, *PME35* and *GAUT7* were upregulated, while that of *PL1* was downregulated. Alongside rising soluble pectin and declining content, pectin degradation dominated over synthesis, with *PG* and *PME35* as the key genes driving protopectin conversion. The expression of all cellulose synthesis-related genes, such as *CESA*2/3, was upregulated in *Z. mioga* flower buds during development. These findings are consistent with the identification of *CESA*2 and *CESA*3 as the genes that control cellulose synthesis in the primary cell wall of *Arabidopsis* [48]. In addition, the cellulose content tended to increase at maturity, suggesting that the higher synthesis than degradation rate led to continuous cellulose accumulation in mature flower buds. Thus, the accumulation of cellulose was attributed to the expression of the *CESA*2 and *CESA*3 genes. Genes responsible for lignin synthesis, such as *Lac*24, peroxidase 6/7/15/47/52 (*POD* 6/7/15/47/52) and *CCR*6 were upregulated, whereas *POD5* and POD*15* were suppressed in the mature GS4 stage. Combined with the increased lignin content between developmental stages (Figure 5E), these upregulated genes (*Lac*24, *POD*6/7/47/52 and *CCR*6) were confirmed to contribute to lignin accumulation.

### 3.3. Correlation Analysis of Polyphenolics and Polysaccharides in the Cell Wall

**Figure 9:**
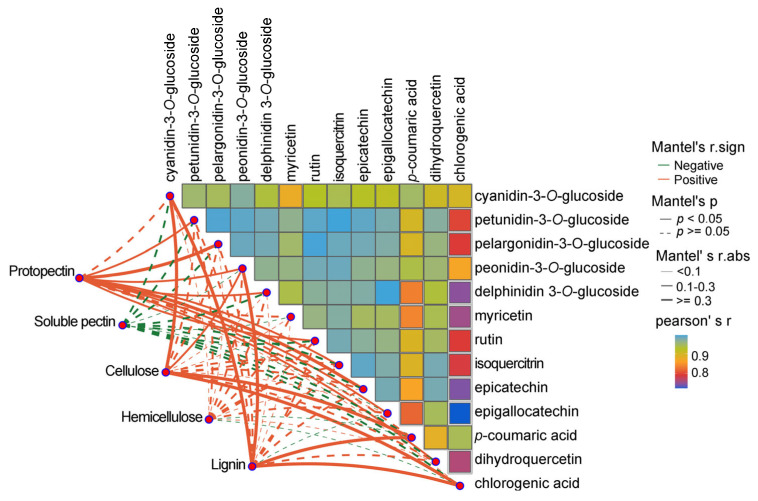
Correlation network heat map analysis between polyphenol compounds and cell wall polysaccharide fractions. Mantel’s r. sign, positive or negative correlation on the basis of the Pearson correlation coefficient; Mantel’s *p*, the *p*-value threshold in the Mantel test; Mantel’s r.abs, the absolute value threshold of correlation between two distance matrices in Mantel’s test; Pearson’s r, Pearson correlation coefficient.

Correlation analysis was performed on data related to the polyphenolic constituents and polysaccharide compounds in the cell wall during growth using Pearson’s correlation coefficients and the Mantel test. The 13 polyphenols shown in Figure 9, whose correlation coefficients were greater than or equal to 0.72, were strongly positively correlated with each other. These results correspond to the anthocyanin synthesis pathway (Figure 4). In this study, no significant differences were detected between most of the cell wall polysaccharides and the polyphenolic compounds (Figure 9). However, significant differences (*p* < 0.05) and positive correlations were detected for protopectin and nine polyphenols (including petunidin-3-*O*-glucoside, pelargonidin-3-*O*-glucoside, peonidin-3-*O*-glucoside, delphinidin 3-*O*-glucoside, rutin, quercetin 3-*β*-*D*-glucoside, epicatechin, epigallocatechin and dihydroquercetin). Positive correlations were also detected for cellulose and lignin with four polyphenols (cyanidin-3-*O*-glucoside, peonidin-3-*O*-glucoside, *p*-coumaric acid and chlorogenic acid). It has been reported in the literature that proanthocyanidins interact with the cell wall and form complexes, with pectin demonstrating the highest affinity for proanthocyanidins among the cell wall polysaccharides [49]. We detected a strong correlation between epicatechin and protopectin (Figure 9). The fact that epicatechin was a product of proanthocyanidin metabolism validated the positive correlation between epicatechin and pectin in our study. Additionally, the positive correlation between lignin and chlorogenic acid was consistent with previous literature, which considered chlorogenic acid to be a reliable intermediate in the lignin biosynthesis pathway [50]. In addition, *p*-coumaric acid, chlorogenic acid and cyanidin-3-*O*-glucoside were strongly positively correlated with cellulose (Figure 9). This, together with the strong correlations between *p*-coumaric acid and lignin/cellulose, is consistent with the findings reported by Chen et al. (2014) [51]. They concluded that coumaric acid is involved in the cross-linking of cellulose and lignin in plant cell walls. In addition, from a metabolic network perspective, polyphenols and cell wall components (such as pectin, cellulose and lignin) have a shared origin in the phenylpropanoid metabolic pathway [52]. Rather than evolving independently, they are subject to a synergistic regulatory relationship involving synthetic competition and non-covalent interactions [53]. This is the biological basis for the high autocorrelation of metabolites and the dominance of a single principal component in explaining their variation in this study (Figure 3). Moreover, from a biological functional perspective, the polyphenols (primarily flavonoids) found in *Z. mioga* flower buds may form stable complexes with pectin and cellulose in the cell wall through hydrogen bonding and hydrophobic interactions [54]. On the one hand, it reduces polyphenol oxidation and browning, while maintaining the buds’ bright colour [55]. On the other hand, modifying the cell wall structure reduces fibre stiffness and enhances tissue water-holding capacity. This ultimately confers sensory characteristics such as crispness, low fibre content and high palatability of the product [56]. This study reveals the aforementioned trends through omics and phenotypic association analyses. However, the specific types of interactions between polyphenols and the cell wall remain unclear. The same is true for the regulatory mechanisms of key genes in metabolic pathways. The relevant conclusions are still based on phenotypic association inferences. Future research should employ in situ binding characterisation and pathway validation to further elucidate the molecular mechanisms and provide more direct theoretical support for improving *Z. mioga* flower bud quality.

## 4. Conclusions

In this study, most polyphenols significantly accumulated during *Z. mioga* development. Cyanidin-3-*O*-glucoside and epicatechin were present at the highest levels throughout the development period. PCA results reveal that PC1 and PC2 accounted for 93.39% and 4.30% of the total variance in metabolites, respectively, indicating that the changes in polyphenol content were largely dependent on the growth and maturation stage and that the germination and maturity stages might represent specific periods during which the contents of polyphenols in *Z. mioga* flower buds change. In addition, the main reason for the increases in the flavonoid and anthocyanin contents may be the upregulation of the expression of *DFR*1/2, *ANS* and *BZl*. Correlation analysis revealed that during development, protopectin was converted to soluble pectin by the synergistic action of PME and PG with *β*-galactosidase; the synergistic interaction of cellulase with PME was involved in cellulose degradation; xylanase was the only enzyme involved in hemicellulose degradation; and Lac interacted synergistically with PME, PG and cellulase for lignin synthesis. The upregulated genes *PME*35, *PG* and *GAUT*7, related to pectin metabolism; *CESA2/3*, related to cellulose synthesis; and *Lac*24, *POD*6/7/47/52 and *CCR*6, related to lignin synthesis were the most important contributors to the changes in cell wall polysaccharides. The 13 polyphenols were strongly positively correlated with each other, with correlation coefficients that were greater than or equal to 0.72. The increased sensory quality of *Z. mioga* flower buds during growth and ripening might be due to the synergistic effects of *p*-coumaric acid, chlorogenic acid, epicatechin, cyanidin-3-*O*-glucoside, peonidin-3-*O*-glucoside, protopectin, pectin, lignin and cellulose. This study helps to further reveal the binding mode of polyphenols and cell wall polysaccharides and provides a theoretical basis for elucidating the formation of the sensory qualities of *Z. mioga* flower buds during growth and maturation.

## Data Availability

The original contributions presented in this study can be found in the paper. For further information, please contact the corresponding author.

## Supplementary Materials

The following supporting information can be downloaded at: https://www.mdpi.com/article/10.3390/metabo16050316/s1, Figure S1: Cluster heat maps of soluble solid, titratable acid and soluble solid/titratable acid in *Z. mioga* flower bud during the development stage. GS1-1/1-2/1-3, the number of repeats at GS1 stage (10 days of growth or germinability); GS2-1/2-2/2-3, the number of repeats at GS2 stage (20 days growing or budding); GS3-1/3-2/3-3, the number of repeats at GS3 stage (30 days growing or late developing); GS4-1/4-2/4-3, the number of repeats at GS4 stage (40 days of growth or ripeness). S/T, the ratio of soluble solid and titratable acid; Figure S2: Overview of the expression pattern of the genes involved in anthocyanin and flavonoid biosynthesis in *Z. mioga* flower buds at different stages. Genes were considered differentially expressed if they both met a ∣log 2-fold change∣ ≥ 1.00 and *p* < 0.05; Figure S3: Overview of the expression pattern of the genes involved in the metabolism of the cell wall in *Z. mioga* flower buds at different stages. Genes were considered differentially expressed if they both met a ∣log 2-fold change∣ ≥ 1.00 and *p* < 0.05; Table S1: Qualitative method for polyphenolics in *Z. mioga* flower bud at different stages. Parent ion, Q1; characteristic fragment ion, Q3; relative molecular mass, M, molecular weight. Each value is the mean of three replicates ± the standard errors; GS1, 10 days of growth or germinability; GS2, 20 days growing or budding; GS3, 30 days growing or late developing; GS4, 40 days of growth or ripeness. Values with different letters for the same polyphenol compound in the table indicate significant differences (*p* < 0.05) in the paired *t*-test; Table S2: The *p* values obtained from the correlation analysis of enzyme activities on the Pearson’s correlation coefficient in *Z. mioga* flower bud with various growth stages. Pectin methylesterase, PME; polygalacturonase, PG; Peroxidase, POD; laccase, Lac; Table S3: Expression levels of genes related to anthocyanin synthesis and cell wall metabolism in the flower buds of *Z. mioga* during development.
