# Supplementary material for: Multomics Analysis of the Characteristic Changes in Polyphenol Accumulation and Cell Wall Polysaccharide Remodelling During the Development of Zingiber mioga Roscoe Flower Buds
Source: Metabolites. 2026 May 8;16(5):316. doi: 10.3390/metabo16050316 (PMC13208317; doi:10.3390/metabo16050316)
Supplement: Supplementary file 1 [file metabolites-16-00316-s001.zip › Supplementary material.pdf]

Supplementary captions

Figure S1: Cluster heat maps of soluble solid, titratable acid and soluble solid/titratable acid in *Z. mioga.* flower bud during the development stage. GS1-1/1-2/1-3, the number of repeats at GS1 stage (10 days of growth or germinability); GS2-1/2-2/2-3, the number of repeats at GS2 stage (20 days growing or budding); GS3-1/3-2/3-3, the number of repeats at GS3 stage (30 days growing or late developing); GS4-1/4-2/4-3, the number of repeats at GS4 stage (40 days of growth or ripeness). S/T, the ratio of Soluble solid and titratable acid.

Figure S2: Overview of the expression pattern of the genes involved in the anthocyanin and flavonoid biosynthesis in *Z. mioga* flower buds at different stages. Genes were considered differentially expressed if they both met a |log 2-fold change| ≥ 1.00 and $P < 0.05$.

Figure S3: Overview of the expression pattern of the genes involved in the metabolism of the cell wall in *Z. mioga* flower buds at different stages. Genes were considered differentially expressed if they both met a |log 2-fold change| ≥ 1.00 and $P < 0.05$.

Table S1: Qualitative method for polyphenolics in *Z*. mioga flower bud at different stages. Parent ion, Q1; characteristic fragment ion, Q3; relative molecular mass, M, molecular weight. Each value was the mean of three replicates ± the standard errors; GS1, 10 days of growth or germinability; GS2, 20 days growing or budding; GS3, 30 days growing or late developing; GS4, 40 days of growth or ripeness. Values with different letters for the same polyphenol compound in the table indicate significant differences ($P < 0.05$) using the paired t-test.

Table S2: The *P* values obtained from the correlation analysis of enzyme activities on the Pearson's correlation coefficient in *Z*. mioga flower bud with various growth stages. Pectin methylesterase, PME; polygalacturonase, PG; Peroxidase, POD; laccase, Lac.

Table S3: Expression levels of genes related to anthocyanin synthesis and cell wall metabolism in the flower buds of *Z. mioga* during development.

Figure S1

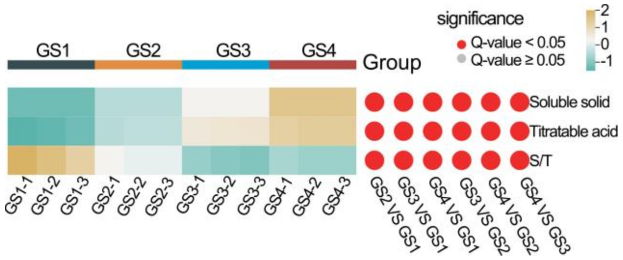

Figure S2

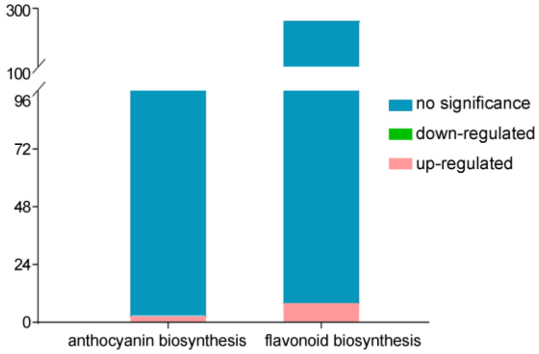

Figure S3

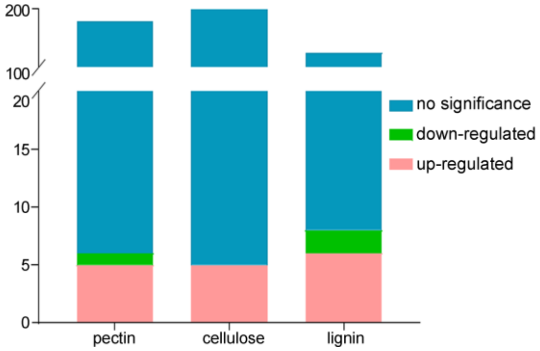

Table S1 Qualitative method for polyphenolics in *Z. mioga* flower bud at different stages.

NO.	Compounds	Identification of the polyphenolic components					Content (μg/g, on dry basis)			
		Q1	Q3	M (Da)	Mode of ionization	CAS	GS1	GS2	GS3	GS4
1	cyanidin-3-*O*-glucoside	449.11	449.11	287.06	7084-24-4	[M]$^{+}$	3.51±0.62^{a}	101.74±4.11^{b}	170.14±26.49 ^{c}	277.52±50.51^{d}
2	petunidin-3-*O*-glucoside	479.12	479.12	317.07	6988-81-4	[M]$^{+}$	0.00±0.00^{a}	0.50±0.04^{b}	1.35±0.13^{c}	3.02±0.22^{d}
3	pelargonidin-3-*O*-glucoside	433.11	433.11	271.06	18466-51-8	[M]$^{+}$	0.30±0.01^{a}	0.51±0.04^{b}	0.81±0.03^{c}	1.47±0.03^{d}
4	peonidin-3-*O*-glucoside	463.12	463.12	301.07	68795-37-9	[M]$^{+}$	0.34±0.07^{a}	3.85±0.06^{b}	9.23±0.21^{c}	16.01±0.93^{d}
5	delphinidin-3-*O*-glucoside	465.10	465.10	303.05	50986-17-9	[M]$^{+}$	0.00±0.00^{a}	1.27±0.25^{b}	9.53±1.40^{c}	25.83±3.41^{d}
6	myricetin	318.04	318.04	153.80	529-44-2	[M+H]$^{+}$	0.00±0.00^{a}	0.00±0.00^{a}	0.12±0.02^{b}	0.24±0.05^{c}
7	rutin	610.15	609.15	301.00	153-18-4	[M+H]$^{+}$	1.02±0.07^{a}	7.54±1.02^{b}	21.12±1.56^{c}	43.84±3.42^{d}
8	isoquercitrin	464.10	463.09	300.03	21637-25-2	[M-H]$^{-}$	0.20±0.01^{a}	0.43±0.07^{b}	0.83±0.04^{c}	1.64±0.18^{d}
9	epicatechin	290.08	291.09	139.04	490-46-0	[M+H]$^{+}$	6.33±0.37^{a}	59.07±7.92^{b}	108.06±2.34^{c}	313.39±32.19^{d}
10	epigallocatechin	306.07	305.07	125.00	970-74-1	[M-H]$^{-}$	1.00±0.06^{a}	1.43±0.11^{b}	6.61±0.32^{c}	18.12±2.37^{d}
11	*p*-coumaric acid	164.05	165.05	119.00	501-98-4	[M+H]$^{+}$	0.99±0.08^{a}	1.44±0.06^{b}	1.63±0.02^{c}	1.86±0.04^{d}
12	dihydroquercetin	304.06	303.05	125.06	480-18-2	[M+H-H_2O]$^{+}$	0.10±0.02^{a}	0.14±0.01^{b}	0.16±0.01^{c}	0.26±0.03^{d}
13	chlorogenic acid	354.10	353.09	191.01	327-97-9	[M-H]$^{-}$	0.44±0.03^{a}	0.54±0.03^{b}	0.61±0.01^{c}	0.62±0.03^{c}

Table S2 The P values obtained from the correlation analysis of enzyme activities on the Pearson's correlation coefficient in *Z. mioga* flower bud with various growth stages.

Enzymes	cellulase	Lac	PG	PME	POD	xylanase	β-galactosidase
cellulase	0.000	0.000	0.005	0.005	0.209	0.168	0.000
Lac	0.000	0.000	0.005	0.004	0.181	0.183	0.000
PG	0.005	0.005	0.000	0.249	0.991	0.952	0.006
PME	0.005	0.004	0.249	0.000	0.554	0.487	0.004
POD	0.209	0.181	0.991	0.554	0.000	0.000	0.225
xylanase	0.168	0.183	0.952	0.487	0.000	0.000	0.250
β-galactosidase	0.000	0.000	0.006	0.004	0.225	0.250	0.000

Table S3 Expression levels of genes related to anthocyanin synthesis and cell wall metabolism in the flower buds of *Z. mioga* during development.

Gene ID	KEGG	EC	Description	Gene expression								
				GS1-1	GS1-2	GS1-3	GS2-1	GS2-2	GS2-3	GS3-1	GS3-2	GS3-3
LOC122034217	00360;00130;00940	EC:6.2.1.12	4-coumarate-CoA ligase 3	0.80	1.11	0.90	5.51	3.96	5.01	66.72	63.71	51.83
LOC122017888	00941;04712	EC:2.3.1.74	chalcone synthase 5	0.88	1.04	1.62	10.11	6.07	7.61	41.13	49.41	53.22
LOC121986029	00941	EC:1.14.11.9	flavanone 3-hydroxylase1	1.32	1.36	1.78	7.47	5.09	4.36	43.92	31.74	33.48
LOC122038526	00941	EC:1.14.11.9	flavanone 3-hydroxylase2	0.60	0.25	0.33	1.29	2.22	1.43	4.83	4.99	5.66
LOC121989454	00941;00944	EC:1.14.14.81	flavonoid 3',5'-hydroxylase 1-like	0.00	0.00	0.00	0.20	0.19	0.27	1.09	1.27	1.00
LOC121989088	00941	EC:1.14.20.6	flavonol synthase 1	0.00	0.00	0.00	3.35	2.21	1.87	31.99	23.26	20.15
LOC122041130	00941	EC:1.14.20.6	flavonol synthase 2	1.50	1.62	2.82	4.81	4.75	6.26	20.96	34.44	22.22
LOC122016967	00941	EC:1.14.20.4	anthocyanidin synthase	0.00	0.00	0.00	0.59	0.44	0.25	6.01	5.92	6.41
LOC122009647	00941	EC:1.1.1.219	dihydroflavonol 4-reductase 1	0.81	0.67	0.78	3.01	3.29	3.73	31.22	44.77	33.48
LOC121971619	00941	EC:1.1.1.219	dihydroflavonol 4-reductase 2	0.43	0.82	1.09	4.51	3.79	5.85	34.97	40.28	38.97
LOC122008556	00944	EC:2.4.1.159	anthocyanidin 3-O-glucosyltransferase	0.10	0.00	0.03	0.68	1.03	0.56	14.00	16.92	12.19
LOC105769889	00940	EC:1.11.1.7	peroxidase 15-like	1.67	1.41	1.36	0.94	1.44	1.14	0.06	0.02	0.04
LOC122056737	00040	EC:4.2.2.2	pectate lyase 1	6.70	8.37	5.86	3.14	2.15	4.60	0.44	0.23	0.15
LOC122008200	00940	EC:1.11.1.7	peroxidase 5	21.96	31.17	28.47	5.45	7.07	6.70	3.30	4.36	3.95
LOC121972066	0052	EC:2.4.1.43	Probable galacturonosyltransferase 7 isoform X3	2.64	3.66	5.76	6.35	5.71	7.04	38.80	22.54	32.05

LOC103990187	NA	NA	laccase-24-like	0.01	0.02	0.04	0.82	0.81	1.00	1.41	1.07	1.20
LOC123413070	NA	NA	cellulose synthase-like protein D2	2.07	3.29	3.53	3.82	3.25	2.44	23.79	17.95	33.50
LOC122008781	0050	EC:3.2.1.4	endoglucanase 7-like isoform X1	0.53	0.35	0.70	1.31	1.13	1.16	6.10	4.10	5.07
LOC122014305	NA	NA	cellulose synthase-like protein D2	3.43	4.75	4.08	5.65	4.14	5.39	46.16	35.63	41.87
LOC121971832	00940	EC:1.11.1.7	peroxidase P7-like	0.02	0.06	0.20	1.15	0.89	0.95	5.01	5.37	3.43
LOC122047065	00940	EC:1.2.1.44	cinnamoyl-CoA reductase-like 6	0.38	0.84	2.10	3.54	1.91	1.67	20.05	17.18	14.09
LOC104897257	00940	EC:1.11.1.7	peroxidase P7-like	0.14	0.06	0.26	0.87	0.77	0.50	3.31	3.67	4.68
LOC135640853	00940	EC:1.11.1.7	peroxidase 52-like	9.32	12.39	24.80	92.73	83.41	58.31	200.98	165.45	179.59
LOC103453544	00040	EC:3.1.1.11	pectinesterase-like	0.37	0.53	0.58	0.70	0.91	1.09	21.29	29.14	36.98
LOC122053059	00940	EC:1.11.1.7	peroxidase 47-like	0.03	0.11	0.16	0.87	0.95	1.54	7.48	10.33	6.27
LOC122021095	00040	EC:3.2.1.67	polygalacturonase-like	0.05	0.01	0.04	0.34	0.53	0.22	13.73	20.95	14.08

NA, not identified.
